# Physician Feedback Reduces Antibiotic Prescribing for Uncomplicated Upper Respiratory Tract Infection in the Emergency Department

**DOI:** 10.3390/antibiotics14121196

**Published:** 2025-11-25

**Authors:** Huiling Guo, Seema Aithal, Hwee Pin Phua, Win Sen Kuan, Eillyne Seow, Yanyi Weng, Hoon Chin Lim, Li Lee Peng, Hann Yee Tan, Angela Chow

**Affiliations:** 1 Department of Epidemiology and Preventive Medicine, Office of Clinical Epidemiology, Analytics, and Knowledge, Tan Tock Seng Hospital, 11 Jalan Tan Tock Seng, Singapore 308433, Singapore; 2 Department of Emergency Medicine, National University Hospital, Singapore 119074, Singapore; win_sen_kuan@nuhs.edu.sg (W.S.K.);; 3 Department of Surgery, Yong Loo Lin School of Medicine, National University of Singapore, Singapore 117597, Singapore; 4 Acute and Emergency Care Department, Khoo Teck Puat Hospital, Singapore 768828, Singaporetan.hann.yee@nhghealth.com.sg (H.Y.T.); 5 Department of Emergency Medicine, Tan Tock Seng Hospital, Singapore 308433, Singapore; yanyi.weng@nhghealth.com.sg; 6 Department of Emergency Medicine, Changi General Hospital, Singapore 529889, Singapore; 7 Lee Kong Chian School of Medicine, Nanyang Technological University, Singapore 639798, Singapore; 8 Saw Swee Hock School of Public Health, National University of Singapore, Singapore 117549, Singapore

**Keywords:** antimicrobial stewardship, emergency department, physician feedback, patient education, inappropriate prescribing

## Abstract

**Objectives:** Inappropriate antibiotic prescribing for acute upper respiratory tract infections (URTIs) is a significant problem in emergency departments (EDs) worldwide. In this study, we separately evaluated the effectiveness of physician feedback and patient education in reducing antibiotic prescribing for URTIs in the ED setting. **Methods:** We conducted a quasi-experimental study across four large adult EDs in Singapore from January 2021 to December 2023. After a baseline period (18 months), two EDs were randomly assigned to either implement physician feedback or patient education for six months (intervention period 1), and all EDs subsequently implemented both interventions in the next six months (intervention period 2). Hierarchical segmented logistic regression was used to assess the effectiveness of the interventions in reducing weekly antibiotic prescribing for URTIs. **Results:** In the two EDs that implemented physician feedback first, there was a significant decrease in the trend of weekly antibiotics prescribed (AOR 0.981 [95% CI (0.964, 0.998)]) compared to the last 6 months of the pre-intervention period. Adding patient education in the next phase (intervention period 2) did not further reduce the trend of weekly antibiotics prescribed. For the second group of two EDs that implemented patient education first, there was no significant reduction in the weekly antibiotic prescribing trend during intervention period 1. With the addition of physician feedback, a significant decline in the trend of weekly antibiotics prescribed was observed in intervention period 2 (AOR 0.921 [95% CI (0.903,0.940)]). **Conclusions:** Physician feedback alone can reduce antibiotic prescribing for URTIs in EDs. Further research is warranted to assess the effectiveness of patient education involving multi-modal educational channels co-created with patients.

## 1. Introduction

Inappropriate use of antibiotics is a major driver of antimicrobial resistance (AMR) [1]. In the emergency departments (EDs), inappropriate antibiotic prescribing is a significant concern, with 27.6–34.2% of all antibiotic prescriptions deemed unnecessary [2,3,4]. Upper respiratory tract infections (URTIs), often viral in origin and unresponsive to antibiotic treatment, represent one of the leading medical conditions associated with inappropriate antibiotic prescribing in the ED setting [5]. Past studies have reported that nearly a quarter of ED visits for antibiotic-inappropriate respiratory diagnoses were prescribed antibiotics [6,7]. Despite EDs exhibiting higher inappropriate antibiotic prescribing rates compared to primary care settings (approximately 9%) [8], antimicrobial stewardship (AMS) programs in the EDs remain underdeveloped [9]. This gap persists even in countries where stewardship efforts are well-established and rigorously enforced across inpatient wards in all acute care public hospitals [10].

The fast-paced ED setting poses unique challenges to the implementation of AMS due to high patient turnover, frequent staff changes, uncertain diagnoses, and limited consultation time [11]. Factors influencing antibiotic prescribing for URTIs in the ED include physician knowledge gaps and patients’ limited understanding of antibiotic use and antibiotic expectations [12,13,14,15]. While the number of AMS interventions specific for EDs may be limited [16,17], successful strategies to reduce inappropriate antibiotic prescribing for URTIs have been highlighted in the literature [18,19,20,21].

In a clinical trial conducted in 16 hospitals, Metlay et al. [18] observed a 10% reduction in antibiotic prescriptions for acute respiratory infections when an intervention package of clinician feedback and education along with patient educational materials was implemented, compared to a 0.5% increase in control sites. Similarly, Yadav et al. [19] reported a significant reduction in inappropriate antibiotic prescribing from 2.2% to 1.5% with an odds ratio of 0.67 (95% CI: 0.54–0.82) after implementing a multifaceted intervention involving patient and physician education, physician champions, departmental feedback, and peer comparison in a pragmatic cluster-randomized clinical trial. To date, the majority of AMS interventions have been multifaceted in nature or involve a bundled approach, which can be difficult to sustain and costly to deliver [22]. This calls for further research that can identify the specific intervention component that is effective on its own [9]. Furthermore, multicenter studies are warranted to identify optimal AMS interventions in the ED setting [11].

In this study, we separately evaluated the effectiveness of physician feedback and patient education on reducing antibiotic prescribing for URTIs in multiple EDs.

## 2. Results

In the pre-intervention periods 1 and 2, a total of 12,794 and 4233 patient attendances for URTIs were managed by 521 and 376 physicians, respectively, at the four EDs (Table 1). During intervention periods 1 and 2, a total of 5445 and 5933 URTI attendances were managed by 444 and 467 physicians, respectively. In the post-intervention observation period, 5322 URTI attendances were managed by 463 physicians.

**Figure 1:**
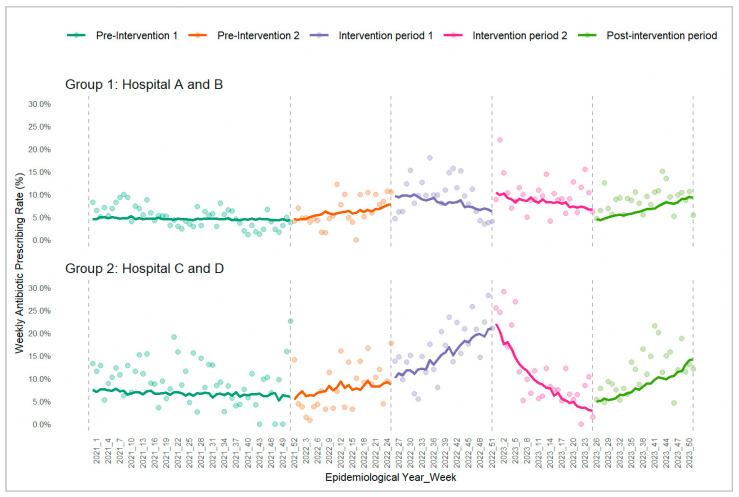
Trends in weekly antibiotic prescribing rate for the two groups across the 5 study periods. Group 1 started with physician feedback; group 2 started with patient education; colored observed points and fitted lines reflect weekly antibiotic prescribing rates and model-derived predicted probabilities, respectively.

To summarize the overall level within each period, the weekly proportions of URTI attendances with antibiotics prescribed were averaged across the weeks in each period (Table 1). For EDs in group 1 (randomized to implement physician feedback in intervention period 1), the mean (SD) was 5.0% (2.3%) and 6.3% (3.2%) in the pre-intervention periods 1 and 2, 9.6% (4.1%) and 10.0% (3.8%) in intervention periods 1 and 2, and 8.8% (2.7%) in the post-intervention period. For EDs in group 2 (randomized to implement patient education in intervention period 1), the corresponding values were comparatively higher at 9.2% (4.8%), 8.2% (5.0%), 16.4% (5.8%), 11.2% (7.9%), and 11.3% (4.7%), respectively. While these mean weekly proportions summarized at the period level provide a useful descriptive overview, they do not capture the week-to-week trajectory or the direction of change within each period. Periods with similar averages can still exhibit markedly different underlying trends. To better reflect temporal patterns relevant to the slope-change estimates, Figure 1 shows the observed weekly antibiotic prescribing rates (depicted as colored dots) together with the fitted predicted probabilities from the hierarchical segmented logistic regression model for both groups across the study periods. The fitted lines illustrate the estimated level and trend (slope) changes at each breakpoint, while the points depict the observed underlying week-to-week variation.

Table 2 summarizes the model estimates, adjusted odds ratios [AORs], and their 95% confidence intervals [CIs] for the level and trend changes, corresponding to the patterns of the fitted lines in Figure 1. A level-change AOR reflects the immediate shift in log-odds at each breakpoint (AORs > 1 indicate an upward shift; AORs < 1 a downward shift). A trend (slope)-change AOR describes how the weekly trend of prescribed antibiotics changes relative to the preceding period, where AOR > 1 indicates that the post-breakpoint trend has become more positive, while AOR < 1 indicates that the trend has become less positive or more negative. Trend-change AOR can also signal a reversal in trend direction—an AOR > 1 may point to a declining pre-breakpoint trend shifting into an upward trajectory, whereas an AOR < 1 may indicate a rising trend reversing into a decline. As the primary interest of our study was in assessing whether the interventions altered the ongoing weekly trajectory of antibiotics prescribed, interpretation of the model results below focuses on the trend-change AORs in the intervention and post-intervention periods relative to the period preceding them for both ED groups.

In group 1 EDs, there was a significant reduction in the trend of weekly antibiotics prescribed (AOR 0.981, 95% CI 0.964, 0.998) after the implementation of physician feedback during intervention period 1 as compared to the prior 6 months of the pre-intervention period. This aligns with the fitted line in Figure 1, which shows a transition from a rising trajectory in pre-intervention period 2 to a declining trajectory in intervention period 1. However, the addition of patient education to physician feedback during intervention period 2 did not result in a further statistically significant decline in the trend of weekly antibiotics prescribed (AOR 0.987, 95% CI 0.970, 1.004). In the post-intervention observation period, there was a significant increase in the weekly antibiotic prescribing trend relative to intervention period 2 (AOR 1.034, 95% CI 1.014, 1.054), as evidenced by a reversal in the fitted trend towards an upward trajectory in Figure 1.

In group 2 EDs, there was a significant increase in the weekly antibiotics prescribing trend (AOR 1.036, 95% CI 1.019, 1.053) after the implementation of patient education during intervention period 1 relative to pre-intervention period 2, consistent with the steeper fitted trend rise seen in Figure 1**.** With the addition of physician feedback to patient education, a significant downward shift in the weekly antibiotic prescribing trend was observed in intervention period 2 (AOR 0.921, 95% CI 0.903, 0.940) compared to intervention period 1; this is visually reflected by the reversal in trajectory in Figure 1. Subsequently, the post-intervention observation period showed a significant increase in the weekly antibiotic prescribing trend compared with intervention 2 (AOR 1.051, 95% CI 1.030, 1.072), reflected by a reversal in the fitted trend towards an upward trajectory in Figure 1.

The results were very similar across the restricted physician groupings following sensitivity analyses (Supplementary Table S1).

## 3. Discussion

The present study evaluated two distinct approaches—physician feedback and patient education—to determine their respective effectiveness in reducing antibiotic prescribing for uncomplicated URTIs in the ED setting. Our findings revealed a significant reduction in the trend of weekly antibiotic prescribing for URTIs in EDs following the implementation of individualized physician feedback that was benchmarked against departmental prescribing norms. Previous randomized controlled trials have demonstrated the effectiveness of audit and feedback interventions in reducing antibiotic prescribing, but these were largely conducted in primary care settings [23]. A pre-COVID-19 controlled before-after study conducted in five adult EDs established the potential of behavioral feedback with peer comparison for ED physicians in reducing inappropriate antibiotic prescribing for URTIs [20]. Our study has further strengthened the evidence for physician feedback as a vital AMS intervention strategy in the ED setting, while taking into consideration the characteristics of patients and physicians.

During the initial pre-intervention period (January 2021–December 2021), we observed a slight decline in the weekly antibiotic prescribing rates across both groups. This trend likely reflected the Delta wave of the COVID-19 pandemic in Singapore, when EDs experienced unprecedented high URTI case volumes due to reduced primary care utilization [24]. The surge in attendance created an artifactually low prescribing rate, as many patients presented for certification of non-infectious status before returning to work. In the latter part of the pre-intervention period (January 2022 to June 2022), a rising trend in the weekly antibiotic prescribing was noted due to the declining ED URTI attendances, which coincided with increased primary care utilization and national policy changes such as the Home Recovery Program, which enabled individuals to self-test and recover at home [25,26]. This upward trend continued into intervention period 1 as COVID-19 restrictions eased and URTI presentations returned to the typical patterns, with the case volumes normalizing [27,28]. This mirrored the rising antibiotic utilization patterns observed in Singapore’s primary care setting [29]. Furthermore, clinical management of COVID-19 discouraged antibiotic use for mild to moderate cases, aligning with recommendations for other URTIs [30]. Therefore, our findings should be interpreted within the context of the post-pandemic period in a tropical setting, where influenza seasonality is not well-defined.

Our results also showed a significant increase in the weekly antibiotic prescribing trends during the post-intervention observation period following the cessation of the combined intervention in both groups, indicating a lack of persistent intervention effect. Past studies in the primary care settings have also demonstrated that improvements in prescribing practices are not sustained without continued intervention efforts [31,32]. This suggests that effective antimicrobial stewardship requires ongoing, active efforts to sustain initial improvements in prescribing behavior.

The success of the physician feedback intervention implemented in our study could be attributed to several factors. A systematic review conducted by Xu et al. [23] highlighted the importance of the dosing frequency of social norm feedback, with monthly feedback having greater effects on antibiotic prescribing rates as compared to feedback given at quarterly or annual intervals. In our study, physician feedback comprising a personalized message was sent by a senior ED physician at two-monthly intervals, thus aligning with the recommended frequency. Furthermore, the framing of the message and the mode of delivery of the feedback were informed by the findings from a prior qualitative study with ED physicians (unpublished). This ensured that the feedback messages were tailored to meet physicians’ specific preferences and requirements. The careful design and delivery of these feedback messages, together with evidence-based tips to reduce antibiotic prescribing for URTIs, could have contributed to the effectiveness of the feedback in reducing antibiotic prescribing rates in this study. Further studies that elucidate the effectiveness of the frequency, mode of delivery, and types of guidance in physician audit and feedback in reducing antibiotic prescribing are warranted.

Conversely, we found that patient education using information leaflets had no impact on antibiotic prescribing rates for URTI patients presenting to the ED, despite the apparent knowledge gaps regarding antibiotic use and AMR in this patient population [33]. Our previous study found that patients with poor knowledge of antibiotics and AMR attending the ED for URTIs were twice as likely to expect antibiotics, whilst patients who expected antibiotics were 10 times more likely to receive them [33]. The lack of effectiveness in patient education utilizing only educational leaflets in our study suggests the need for multi-modality in education.

The passive dissemination of educational leaflets to patients without accompanying explanations from healthcare providers has been shown to have little impact on reducing antibiotic prescribing rates. These materials are more effective when used as tools for shared decision-making between patients and healthcare providers [34,35]. Additionally, the educational leaflet used in the present study was developed solely by the study team, potentially missing key messages that patients would find relevant and meaningful in understanding appropriate antibiotic use and AMR [36]. Antibiotic use behaviors exhibited by URTI patients are not homogeneous. Previous experiences with both URTIs and antibiotics influence how different patients assess their need for and expectations around antibiotic treatment [37]. Overall, these findings highlight the need for future patient education interventions that acknowledge and appreciate the heterogeneous nature of URTI patients while involving different patient groups in the co-creation of educational materials to improve the content and language that effectively promote desirable antibiotic behaviors [38,39]. Future research should collaborate with patients to identify the optimal moments in the ED journey where educational materials can be most effectively delivered to facilitate meaningful discussions on the appropriate use of antibiotics for URTIs between patients and physicians.

The strength of our study lies in the use of a quasi-experimental study design to assess the effectiveness of physician feedback and patient education, separately and in combination, in reducing antibiotic prescribing for URTIs. Our findings demonstrate the impact of a simple low-resource AMS intervention like physician feedback on clinical outcomes, albeit marginally, for uncomplicated URTIs in the ED. These results contribute to the limited evidence base on effective AMS strategies in ED settings. Additionally, our study utilizes robust modeling to illustrate prescribing trends rather than relying on simple pre–post comparisons, which enhances the rigor and interpretability of our analyses.

We acknowledge that our study could be limited by potential confounding, which was not measured due to the use of secondary data obtained from clinical notes. Nonetheless, the use of multivariable multi-level hierarchical regression models has addressed potential clustering and confounding effects due to physician and hospital factors. The use of hierarchical model specification also helps to mitigate potential confounding from time-related variations by allowing each physician’s prescribing tendency to serve as their own reference over time. Furthermore, the outcome measure of interest was based on the actual antibiotic prescribed, eliminating any classification bias. Although our interventions were standardized across all four participating hospitals and physician feedback was systematically delivered at appropriate intervals, we cannot confirm that informational leaflets were consistently distributed to URTI patients prior to their ED physician consultations, despite regular reminders to nursing staff about study protocols and distribution procedures. Finally, we recognize the lack of a comparator group for contemporaneous comparison; however, the assessment of consecutive six-monthly periods likely balances any minor seasonal influences between the pre-intervention and intervention phases.

## 4. Methods

### 4.1. Study Design and Settings

**Figure 2:**
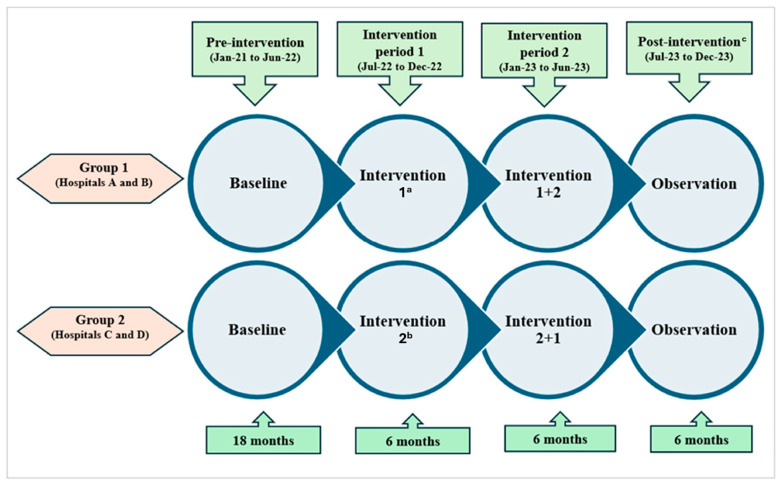
Schematic diagram of the quasi-experimental study of 2 tailored antimicrobial stewardship interventions conducted in 4 emergency departments. ^a^ Intervention 1—physician feedback; ^b^ intervention 2—patient education; ^c^ all interventions discontinued in this phase for both groups.

From January 2021 to December 2023, a quasi-experimental study was conducted in four large adult EDs in Singapore. The study comprised four phases: (1) ***pre-intervention period*** for 18 months (January 2021 to June 2022); (2) ***intervention period 1*** for 6 months (July 2022 to December 2022) when two EDs (group 1: hospitals A, B) were randomly assigned to implement physician feedback and the other two EDs (group 2: hospitals C, D) to patient education; (3) ***intervention period 2*** for 6 months (January 2023 to June 2023) when all EDs implemented both physician feedback and patient education; and (4) ***post-intervention observation period*** for the last 6 months (July 2023 to December 2023) when all interventions were discontinued (Figure 2). The study protocol has been described in detail in a separate publication [40].

Singapore’s emergency healthcare system comprises both public restructured hospitals and private healthcare facilities, with public sector ED visits accounting for 84% of the total patient volume. The total ED beds and median number of annual ED attendances for the 9 public sector hospitals in 2021 were 555 and 105,752, respectively [41]. The two EDs in group 1 were Changi General Hospital (CGH) and Khoo Teck Puat Hospital (KTPH), whilst group 2 included EDs from Tan Tock Seng Hospital (TTSH) and National University Hospital (NUH). All four EDs are part of academic teaching hospitals catering to a rapidly aging population.

This study was approved by the Domain Specific Review Board, National Healthcare Group Singapore (reference number: 2019/00174, dated 11 September 2019), and registered on ClinicalTrials.gov (reference number: NCT05451836).

### 4.2. Interventions

For EDs implementing physician feedback, a personalized message was sent at two-monthly intervals by a senior ED physician to individual ED physicians on his/her individual antibiotic prescribing rate for uncomplicated URTIs, alongside the departmental average in the past month, to show their performance relative to their peers. The feedback was delivered via a protected messaging platform (TigerConnect, Inc., Santa Monica, CA, USA) used by public hospitals in Singapore. Each message also included concise, evidence-based tips for reducing antibiotic prescribing in URTI cases tailored to the local setting, based on input from senior ED physicians at the study sites. Three feedback messages were delivered during the 6-month intervention period, commencing after the first month.

On the other hand, for EDs implementing patient education, locally contextualized patient information leaflets (in the preferred language—English, Chinese, Malay, or Tamil) were distributed to patients with URTIs by the ED triage nurse prior to their consultation with the physician. The leaflets were developed based on patient educational materials from the United States Centers for Disease Control and Prevention, with modifications for the local ED setting and a readability grade level of 7. The study team conducted regular visits to each ED to reinforce nursing staff about adherence to the study protocol and distribution procedures and to ensure that patient education leaflets were consistently available and displayed. An example of the personalized physician feedback message and the English version of the patient leaflet are available in the Supplementary Materials.

### 4.3. Data Analysis

The unit time used in data analysis was the epidemiological week. The period used in analysis spanned a total of 156 weeks and was divided into five consecutive segments of 26 weeks except for the first segment, which comprised 52 weeks. The pre-intervention period was segmented into two to facilitate the comparison of trends across periods of comparable duration. These were demarcated by weeks 1–52 (pre-intervention period 1), weeks 53–78 (pre-intervention period 2), weeks 79–104 (intervention period 1), weeks 105–130 (intervention period 2), and weeks 131–156 (post-intervention period).

The URTI case definition for our study included ED visits with a primary diagnosis of acute URTIs (International Classification of Diseases, 9th Revision, Clinical Modification [ICD-9-CM] code: 465 or [ICD-10-CM code: J06.9] denoting acute URTI, unspecified) among patients who were discharged from the ED. The analysis included only physicians with more than one URTI patient visit during the above-defined period (N = 1249).

Characteristics of attending physicians and URTI visits, and the proportion of antibiotics prescribed in each week during pre-intervention period 1, pre-intervention period 2, intervention period 1, intervention period 2, and post-intervention period for the two groups of hospitals were summarized using frequencies and percentages or mean and standard deviation (SD) where appropriate.

We developed a hierarchical (mixed-effects) segmented logistic regression model with log odds of antibiotic prescribing as the outcome to estimate the impact of the intervention on the trend (slope) and level (intercept) over time for the two groups of hospitals. In the patient visit level, time was modeled as a continuous variable in epidemiological week with piecewise linear splines introduced at each of the pre-intervention, intervention, and post-intervention breakpoints (weeks 52, 78, 104, and 130, respectively) to capture changes in time trends (slope). Binary indicator variables were included to estimate the level of changes at the start of each intervention only. Interaction terms between hospital groups and (a) time segments and (b) the intervention indicator variables were added to evaluate effects of the intervention across the two hospital groups. The analysis accounted for the hierarchical data structure with patient visits nested within their attending physicians, who in turn were nested within hospitals. Random intercepts were included at both the physician and hospital levels to account for this clustering. We included physician years of clinical experience and patient characteristics (age group, gender, ethnicity) decided a priori to be potential confounders of antibiotic prescribing [4,42,43,44]. Singapore’s tropical location leads to year-round circulation of influenza viruses, with bimodal peaks typically occurring in May–July (Southern Hemisphere season) and December–March (Northern Hemisphere season). Therefore, we did not adjust for seasonal differences due to the absence of a well-defined influenza season.

The above modeling approach allowed the estimation of the following for each group of hospitals: (a) the immediate change in weekly antibiotic prescribing level at intervention onset (Weeks 79 and 105); (b) the change in weekly prescribing trend following intervention; and (c) sustained effects in the post-intervention period. As the interventions were expected to influence the weekly prescribing trend rather than produce large instantaneous shifts, slope-change parameters during the intervention and post-intervention periods would be the primary focus of interest, with the level-change terms included at intervention breakpoints to allow modeling flexibility. The likelihood ratio test was used to compare the three-level hierarchical logistic regression model with a two-level model that included a random intercept for physicians only. Observed weekly antibiotic prescribing rates and fitted predicted probabilities from the regression model were plotted to illustrate the estimated temporal patterns. Details on the model are provided in the Supplementary Materials (Supplementary Table S2). To explore the robustness of the findings, sensitivity analyses were also conducted on (1) physicians who attended to URTI patients in the pre-intervention and at least one out of two intervention periods; and (2) physicians with data in the pre-intervention and both intervention periods. Statistical analyses were performed using STATA version 18.0 (StataCorp LLC, College Station, TX, USA), and the plot of observed and model-fitted probabilities was produced in R version 4.4.0 (R Foundation for Statistical Computing, Vienna, Austria).

## 5. Conclusions

Physician feedback alone can reduce antibiotic prescribing for URTIs in EDs. However, patient education using information leaflets developed by health professionals did not reduce antibiotic prescribing. Future research is warranted to assess the effectiveness of patient education in reducing antibiotic prescribing in the ED. The co-creation of multi-modal educational channels with patients—especially those that can facilitate patient–physician discussions on antibiotics—should be considered.

## Figures and Tables

**Table 1:** Characteristics of patient attendances with uncomplicated upper respiratory tract infections, their attending physicians, and antibiotic prescribing rates, before and following two tailored antimicrobial stewardship interventions conducted in four emergency departments.

		Group 1 (Hospital A and Hospital B)					Group 2 (Hospital C and Hospital D)			
	Pre-Intervention Period 1 (Jan 2021–Dec 2021	Pre-Intervention Period 2 (Jan 2022–Jun 2022)	Intervention Period 1 Intervention 1 ^a^ (Jul 2022–Dec 2022)	Intervention Period 2 Intervention 1 + 2 (Jan 2023–Jun 2023)	Post-Intervention (Jul 2023–Dec 2023)	Pre-Intervention Period 1 (Jan 2021–Dec 2021)	Pre-Intervention Period 2 (Jan 2022–Jun 2021)	Intervention Period 1 Intervention 2 ^b^ (Jul 2022–Dec 2022)	Intervention Period 2 Intervention 2 + 1 (Jan 2023–Jun 2023)	Post-Intervention (Jul 2023–Dec 2023)
**URTI attendance characteristics**										
Number of URTI attendances	8673	2857	3064	3411	2994	4121	1376	2381	2522	2328
Age in years, mean (SD)	33.6 (13.6)	36.7 (16.4)	36.8 (16.4)	39.1 (16.5)	39.1 (17.3)	37.1 (14.5)	38.6 (15.6)	38.2 (15.6)	40.0 (15.8)	40.4 (16.1)
Age group, %										
21–34 years	66.3%	58.9%	58.2%	50.3%	52.1%	55.5%	52.4%	53.2%	47.7%	46.5%
35–49 years	19.9%	19.4%	20.8%	24.9%	22.5%	25.8%	25.4%	25.5%	27.2%	27.5%
50 years and above	13.8%	21.7%	21.0%	24.8%	25.3%	18.7%	22.2%	21.3%	25.1%	26.0%
Gender, %										
Male	60.7%	62.9%	63.3%	57.9%	61.7%	51.8%	52.3%	54.4%	50.0%	47.0%
Female	39.3%	37.1%	36.7%	42.1%	38.3%	48.2%	47.7%	45.6%	50.0%	53.0%
Ethnicity, %										
Chinese	43.7%	41.9%	37.4%	38.0%	39.3%	44.6%	39.5%	42.6%	43.3%	48.0%
Malay	25.0%	23.6%	28.9%	27.6%	25.5%	17.3%	18.2%	19.7%	20.1%	16.5%
Indian	15.6%	16.9%	16.8%	17.1%	17.2%	17.3%	19.2%	19.3%	19.2%	16.4%
Others	15.7%	17.6%	16.8%	17.3%	18.0%	20.8%	23.1%	18.4%	17.3%	19.1%
**Physician characteristics**										
Number of attending physicians	216	171	201	234	225	305	205	243	233	238
Years of clinical experience, mean (SD)	4.7 (4.2)	6.2 (5.6)	5.8 (5.4)	5.9 (5.4)	5.9 (5.5)	6.3 (6.2)	7.6 (6.6)	7.5 (6.5)	7.5 (6.7)	7.3 (6.6)
Years of clinical experience, min–max	0.5–30.4	1.0–31.6	0.4–32.2	0.8–32.7	0.1–33.1	1.0–34.7	1.0–35.2	1.1–35.7	1.0–36.2	0.2–36.7
**Antibiotic prescribing rates per week**										
Number of URTI attendances, mean (SD)	167 (43)	110 (61)	118 (21)	131 (24)	115 (18)	79 (28)	53 (25)	92 (18)	97 (23)	90 (16)
Number of URTI attendances, min–max	95–302	36–260	89–170	87–191	73–162	26–168	20–125	58–132	62–137	62–123
Antibiotic prescribing for URTI attendances (%), mean (SD)	5.0% (2.3%)	6.3% (3.2%)	9.6% (4.1%)	10.0% (3.8%)	8.8% (2.7%)	9.2% (4.8%)	8.2% (5.0%)	16.4% (5.8%)	11.2% (7.9%)	11.3% (4.7%)

^a^ Intervention 1—physician feedback, ^b^ Intervention 2—patient education, SD—standard deviation, URTI—upper respiratory tract infection.

**Table 2:** Hierarchical segmented logistic regression analysis of uncomplicated URTI attendances following two tailored antimicrobial stewardship interventions conducted in four emergency departments.

		All 1249 Physicians with > 1 URTI Patient Attendance During the Study Period Number of Observations: 33,727					
		Estimate	SE	AOR ^a^	Lower Bound 95% CI	Upper Bound 95% CI	*p* Value *
**Group 1** (Hospital A and Hospital B)	**Started with physician feedback**						
	Level at baseline (week 1)	−3.467	0.178	0.031	0.022	0.044	**<0.001**
	Pre-intervention period 1 trend	−0.002	0.001	0.998	0.995	1.001	0.220
	Trend (slope) change in pre-intervention period 2 (relative to pre-intervention period 1)	0.022	0.007	1.023	1.009	1.036	**0.001**
	Level change at the start of intervention period 1	0.897	0.151	2.452	1.825	3.294	**<0.001**
	Trend (slope) change in intervention period 1 (relative to pre-intervention period 2)	−0.019	0.009	0.981	0.964	0.998	**0.032**
	Level change at the start of intervention period 2	0.829	0.166	2.291	1.656	3.169	**<0.001**
	Trend (slope) change in intervention period 2 (relative to intervention period 1)	−0.013	0.009	0.987	0.970	1.004	0.137
	Trend (slope) change in post-intervention period (relative to intervention period 2)	0.033	0.010	1.034	1.014	1.054	**0.001**
**Group 2** (Hospital C and Hospital D)	**Started with patient education**						
	Level at baseline (week 1)	−3.200	0.183	0.041	0.028	0.058	**<0.001**
	Pre-intervention period 1	−0.004	0.002	0.996	0.993	0.999	**0.004**
	Trend (slope) change in pre-intervention period 2 (relative to pre-intervention period 1)	0.022	0.008	1.023	1.007	1.039	**0.005**
	Level change at the start of intervention period 1	0.688	0.162	1.989	1.449	2.731	**<0.001**
	Trend (slope) change in intervention period 1 (relative to pre-intervention period 2)	0.035	0.008	1.036	1.019	1.053	**<0.001**
	Level change at the start of intervention period 2	1.666	0.168	5.289	3.806	7.348	**<0.001**
	Trend (slope) change in intervention period 2 (relative to intervention period 1)	−0.082	0.010	0.921	0.903	0.940	**<0.001**
	Trend (slope) change in post-intervention period (relative to intervention period 2)	0.050	0.010	1.051	1.030	1.072	**<0.001**

Outcome or level, is the log-odds of antibiotic prescribing; trend: log-odds of antibiotic prescribing per week. Pre-intervention period 1: week 1–52; pre-intervention period 2: week 53–78; intervention period 1: week 79–104; intervention period 2: week 105–130; post-intervention period: week 131–156. CI—confidence interval, SE—standard error, AOR—adjusted odds ratio. ^a^ Adjusted for patient age, gender, ethnicity, and physician years of experience; * Bolded values indicate statistical significance of *p* < 0.05. Note: The physician-level (within hospital) intraclass correlation coefficient (ICC) was 0.156, while the hospital-level ICC was 0.011. The likelihood ratio test indicated that a 3-level hierarchical model with random intercept at the hospital and physician levels provided a significantly better fit to the data as compared to a 2-level model with random intercept at the physician-level only (*p* < 0.001).

## Data Availability

The data presented in this study are available on reasonable request from the corresponding author.

## Supplementary Materials

The following supporting information can be downloaded at: https://www.mdpi.com/article/10.3390/antibiotics14121196/s1, Table S1. Sensitivity analysis showing hierarchical segmented logistic regression analysis of uncomplicated URTI attendances following two tailored antimicrobial stewardship interventions conducted in four emergency departments for restricted physician samples. Table S2. Details on model creation.
