# Supplementary material for: Physician Feedback Reduces Antibiotic Prescribing for Uncomplicated Upper Respiratory Tract Infection in the Emergency Department
Source: Antibiotics (Basel). 2025 Nov 25;14(12):1196. doi: 10.3390/antibiotics14121196 (PMC12729386; doi:10.3390/antibiotics14121196)

**Supplementary Table S1 – Sensitivity analysis showing hierarchical segmented logistic regression analysis of uncomplicated URTI attendances following two tailored antimicrobial stewardship interventions conducted in four emergency departments for restricted physician samples.**

|  | **294 physicians with data in the pre-intervention and at least 1 out of 2 intervention periods**  **Number of observations: 21423** | | | | | | **156 physicians with data in the pre-intervention and both intervention periods**  **Number of observations: 18251** | | | | | |
| --- | --- | --- | --- | --- | --- | --- | --- | --- | --- | --- | --- | --- |
|  | **Estimate** | **SE** | **AOR^a^** | **Lower bound 95% CI** | **Upper bound 95% CI** | **P value** | **Estimate** | **SE** | **AOR^a^** | **Lower bound 95% CI** | **Upper bound 95% CI** | **P value** |
| **Group 1 (Hospital A & Hospital B) - Started with physician feedback** | | | | | | | | | | | | |
| Level at baseline (week 1) | -3.326 | 0.194 | 0.036 | 0.025 | 0.053 | **<0.001** | -3.432 | 0.259 | 0.032 | 0.019 | 0.054 | **<0.001** |
| Pre-intervention period 1 trend | -0.003 | 0.002 | 0.997 | 0.993 | 1.000 | 0.070 | -0.003 | 0.002 | 0.997 | 0.993 | 1.001 | 0.119 |
| Trend (slope) change in pre-intervention period 2 (relative to pre-intervention period 1) | 0.024 | 0.008 | 1.024 | 1.009 | 1.040 | **0.002** | 0.017 | 0.009 | 1.017 | 1.000 | 1.035 | 0.051 |
| Level change at the start of intervention period 1 | 0.977 | 0.172 | 2.656 | 1.895 | 3.722 | **<0.001** | 0.978 | 0.183 | 2.659 | 1.858 | 3.804 | **<0.001** |
| Trend (slope) change in intervention period 1 (relative to pre-intervention period 2) | -0.024 | 0.011 | 0.976 | 0.956 | 0.997 | **0.024** | -0.025 | 0.011 | 0.976 | 0.955 | 0.997 | **0.028** |
| Level change at the start of intervention period 2 | 0.856 | 0.213 | 2.355 | 1.552 | 3.572 | **<0.001** | 0.784 | 0.221 | 2.191 | 1.422 | 3.375 | **<0.001** |
| Trend (slope) change in intervention period 2 (relative to intervention period 1) | -0.019 | 0.012 | 0.981 | 0.959 | 1.004 | 0.097 | -0.020 | 0.012 | 0.980 | 0.957 | 1.004 | 0.103 |
| Trend (slope) change in post-intervention period (relative to intervention period 2) | 0.033 | 0.013 | 1.034 | 1.008 | 1.060 | **0.011** | 0.030 | 0.013 | 1.030 | 1.004 | 1.058 | **0.026** |
|  |  |  |  |  |  |  |  |  |  |  |  |  |
| **Group 2 (Hospital C & Hospital D) - Started with patient education** | | | | | | | | | | | | |
| Level at baseline (week 1) | -3.217 | 0.209 | 0.040 | 0.027 | 0.060 | **<0.001** | -3.256 | 0.281 | 0.039 | 0.022 | 0.067 | **<0.001** |
| Pre-intervention period 1 trend | -0.007 | 0.002 | 0.993 | 0.989 | 0.997 | **<0.001** | -0.008 | 0.002 | 0.992 | 0.988 | 0.997 | **0.001** |
| Trend (slope) change in pre-intervention period 2 (relative to pre-intervention period 1) | 0.029 | 0.010 | 1.030 | 1.011 | 1.049 | **0.002** | 0.027 | 0.011 | 1.027 | 1.004 | 1.050 | **0.019** |
| Level change at the start of intervention period 1 | 0.784 | 0.195 | 2.189 | 1.493 | 3.210 | **<0.001** | 0.879 | 0.214 | 2.408 | 1.583 | 3.662 | **<0.001** |
| Trend (slope) change in intervention period 1 (relative to pre-intervention period 2) | 0.043 | 0.010 | 1.044 | 1.025 | 1.064 | **<0.001** | 0.043 | 0.011 | 1.044 | 1.023 | 1.066 | **<0.001** |
| Level change at the start of intervention period 2 | 2.057 | 0.213 | 7.825 | 5.158 | 11.873 | **<0.001** | 2.128 | 0.222 | 8.396 | 5.430 | 12.983 | **<0.001** |
| Trend (slope) change in intervention period 2 (relative to intervention period 1) | -0.094 | 0.013 | 0.910 | 0.887 | 0.934 | **<0.001** | 0.101 | 0.014 | 0.904 | 0.880 | 0.929 | **<0.001** |
| Trend (slope) change in post-intervention period (relative to intervention period 2) | 0.056 | 0.014 | 1.058 | 1.029 | 1.088 | **<0.001** | 0.057 | 0.015 | 1.059 | 1.028 | 1.091 | **<0.001** |
| Outcome or level is the log-odds of antibiotic prescribing; Trend: log-odds of antibiotic prescribing per week  Pre-intervention period 1: week 1-52; Pre-intervention period 2: week 53-78; Intervention period 1: week 79-104; Intervention period 2: week 105-130; Post-intervention period: week 131-156  CI - Confidence Interval, SE – Standard Error, OR - Odds Ratio  ^a^Adjusted for patient age, gender, ethnicity and physician years of experience; * Bolded values indicate statistical significance of p < 0.05.  ^Note: A likelihood ratio test showed that a 3-level hierarchical model with random intercept at hospital and at physician level has a significantly better fit to the data than a 2-level model with random intercept at physician level (p < 0.001) | | | | | | | | | | | | |

**Supplementary Table S2 - Details on model creation**

The hierarchical segmented logistic regression model can be expressed as:

$$\text{log (odds of antibiotic prescribing) =}\text{ }$$

$$\beta_{0}\text{ }\text{+ }\text{ }\beta_{1}\times t_{wk}\text{ + }\beta_{2}\times t_{preI2}\text{ + }\delta_{3}\times I_{I1}+ \beta_{3}\times t_{I1}+ \delta_{4}\times I_{I2}+ \beta_{4}\times t_{I2}+ \beta_{5}\times t_{post}+$$

$\alpha_{0}\times group+ \partial_{1}\times t_{wk}\times group+ \partial_{2}\times t_{preI2}\times group+ \theta_{3}\times I_{I1}\times group+ \partial_{3}\times t_{I1}\times group+ \theta_{4}\times I_{I2}\times group+ \partial_{4}\times t_{I2}\times group+\partial_{5}\times t_{post}\times group+ {\gamma'}_{m}{C'}_{m}+$ $random intercept for physician varying over hospital+random intercept for hospital+residual errors$

where:

| $t_{wk}$ | Time in week since start of study |
| --- | --- |
| $t_{preI2}$ | Pre-intervention period between week 53 and 78: $t_{wk}-52 if {52<t}_{wk}\leq78;0 otherwise$ |
| $t_{I1}$ | Intervention period 1 between week 79 and 104:  $t_{wk}-78 if {78<t}_{wk}\leq104;0 otherwise$ |
| $t_{I2}$ | Intervention period 2 between week 105 and 130  $t_{wk}-104 if {104<t}_{wk}\leq130;0 otherwise$ |
| $t_{post}$ | Post-intervention period between week 131 and 156  $t_{wk}-130, 130<t_{eweek}\leq156$ |
| $I_{I1}$ | Binary indicator of intervention status, first intervention  $1 if {78<t}_{wk}\leq104;0 otherwise$ |
| $I_{I2}$ | Binary indicator of Intervention status, combined interventions  $1 if {104<t}_{wk}\leq130;0 otherwise$ |
| group | Group variable (binary)  1 if Group 1 (Hospital A, B), 0 if Group 2 (Hospital C, D) |
| ${C'}_{m}$ | Set of m visit-level or physician-level observed covariates |

Parameters explanation

| $\beta_{0}$ | Baseline log odds (of prescribing) at week 1 for group 1 |
| --- | --- |
| $\beta_{1}$ | Trend in the pre-intervention period 1 for group 1 |
| $\beta_{2}$ | Change in trend in pre-intervention period 2 as compared to pre-intervention period 1 for group 1 |
| $\beta_{3}$ | Change in trend in intervention period 1 as compared to pre-intervention period 2 for group 1 |
| $\beta_{4}$ | Change in trend in intervention period 2 as compared to intervention period 1 for group 1 |
| $\beta_{5}$ | Change in trend in post-intervention period as compared to intervention period for group 1 |
| $\delta_{3}$ | Level shift in log odds (of prescribing) just at the start of intervention period 1 for group 1 |
| $\delta_{4}$ | Level shift in log odds (of prescribing) just at the start of intervention period 2 for group 1 |
| $\alpha_{0}$ | Group-specific difference in baseline log odds (of prescribing) at week 1 |
| $\partial_{1}$ | Group-specific difference in trend change in pre-intervention period 1 |
| $\partial_{2}$ | Group-specific difference in trend change in pre-intervention period 2 as compared to pre-intervention period 1 |
| $\partial_{3}$ | Group-specific difference in trend change in intervention period 1 as compared to pre-intervention period 2 |
| $\partial_{4}$ | Group-specific difference in trend change in intervention period 2 as compared to pre-intervention period 1 |
| $\partial_{5}$ | Group-specific difference in trend change in post-intervention period as compared to intervention period 2 |
| $\theta_{3}$ | Group-specific difference in the level shift in log odds (of prescribing) at the start of intervention period 1 |
| $\theta_{4}$ | Group-specific difference in the level shift in log odds (of prescribing) at the start of intervention period 2 |
| ${\gamma'}_{m}$ | Set of regression coefficients for the visit-level or physician-level observed covariates |

**Supplementary material – Physician feedback message**


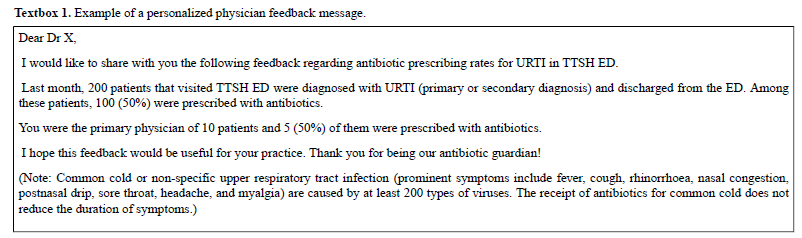

Supplement: Supplementary file 1 [file antibiotics-14-01196-s001.zip › Supplementary materials.docx]
